# Risk of breakthrough infection and hospitalisation after COVID-19 primary vaccination by HIV status in four Italian regions during 2021

**DOI:** 10.1186/s12889-024-19071-y

**Published:** 2024-06-11

**Authors:** Alberto Mateo-Urdiales, Massimo Fabiani, Flavia Mayer, Chiara Sacco, Valeria Belleudi, Roberto Da Cas, Emmanouil Alexandros Fotakis, Luigi De Angelis, Maria Cutillo, Daniele Petrone, Cristina Morciano, Andrea Cannone, Martina Del Manso, Flavia Riccardo, Antonino Bella, Franscesca Menniti-Ippolito, Patrizio Pezzotti, Stefania Spila Alegiani, Marco Massari

**Affiliations:** 1 Department of Infectious Diseases, Istituto Superiore di Sanità, Rome, Italy; 2 National Center for Drug Research and Evaluation, Istituto Superiore di Sanità, Rome, Italy; 3 European Programme on Intervention Epidemiology Training (EPIET), European Centre for Disease Prevention and Control, Stockholm, Sweden; 4 Department of Epidemiology, Lazio Regional Health Service, ASL Roma 1, Rome, Italy; 5 Department of Translational Research and New Technologies in Medicine and Surgery, University of Pisa, Pisa, Italy

**Keywords:** HIV, COVID-19, VACCINES, AIDS

## Abstract

**Background:**

As of 2024, vaccination remains the main mitigation measure against COVID-19, but there are contradictory results on whether people living with HIV (PLWH) are less protected by vaccines than people living without HIV (PLWoH). In this study we compared the risk of SARS-CoV-2 infection and COVID-19 hospitalisation following full vaccination in PLWH and PLWoH.

**Methods:**

We linked data from the vaccination registry, the COVID-19 surveillance system and from healthcare/pharmacological registries in four Italian regions. We identified PLWH fully vaccinated (14 days post completion of the primary cycle) and matched them at a ratio of 1:4 with PLWoH by week of vaccine administration, age, sex, region of residence and comorbidities. Follow-up started on January 24, 2021, and lasted for a maximum of 234 days. We used the Kaplan-Meier estimator to calculate the cumulative incidence of infection and COVID-19 hospitalisation in both groups, and we compared risks using risk differences and ratios taking PLWoH as the reference group.

**Results:**

We matched 42,771 PLWH with 171,084 PLWoH. The overall risk of breakthrough infection was similar in both groups with a rate ratio (RR) of 1.10 (95% confidence interval (CI):0.80–1.53). The absolute difference between groups at the end of the study period was 8.28 events per 10,000 person-days in the PLWH group (95%CI:-18.43-40.29). There was a non-significant increase the risk of COVID-19 hospitalisation among PLWH (RR:1.90; 95%CI:0.93–3.32) which corresponds to 6.73 hospitalisations per 10,000 individuals (95%CI: -0.57 to 14.87 per 10,000).

**Conclusions:**

Our findings suggest PLWH were not at increased risk of breakthrough SARS-CoV-2 infection or COVID-19 hospitalisation following a primary cycle of mRNA vaccination.

**Supplementary Information:**

The online version contains supplementary material available at 10.1186/s12889-024-19071-y.

## Background

As of January 2024, vaccination remains the main measure to reduce the impact of COVID-19 both at the individual and at the population level. In Italy, despite having approved seven different COVID-19 vaccines (BNT162b2, mRNA-1273, ChAdOx1, Ad26.COV2.S, NVX-CoV2373, VLA2001 and VidPrevtyn Beta), over 80% of dose administrations have been from two mRNA vaccines: BNT162b2 (Cominarty) and mRNA-1273 (Moderna) [1]. The efficacy of these vaccines was demonstrated in experimental studies [2–6], and several observational studies have corroborated their effectiveness post-authorisation [7–9]. However, multiple viral and individual factors have been found to influence vaccine-induced protection, such as the emergence of more transmissible SARS-CoV-2 variants [8]. The immune status is another factor that has been described to impact on the individual response to the vaccine, with several studies finding a lower immune response in immunocompromised persons, such as recipients of solid organ transplants [10–12].

People living with HIV (PLWH) are among those at higher risk of having immune dysfunction [13]. In Italy, the incidence of newly diagnosed cases of PLWH has been decreasing over the last decade, with an annual incidence of 2.2 cases per 100,000 inhabitants in 2020 [14], and over 90% of PLWH on antiretroviral therapy (ART), which reduces the risk of immune dysfunction [14]. However, the number of people diagnosed at a late stage of infection -i.e. those with a low CD4 count- has been increasing in the last years [15]. Thus, many PLWH may still be at risk of immune dysregulation, which may increase the likelihood of developing severe COVID-19 and, at the same time, it could impact negatively the protection conferred by COVID-19 vaccines. In this sense, previous studies have found a poorer humoral and cell-mediated response against SARS-CoV-2 following mRNA vaccination in PLWH with < 200 per mm^3^ CD4 T-cells, whilst the immunity response in those with a high CD4 count (> 500 mm^3^) was comparable to the HIV negative population [16].

However, few studies have compared effectiveness of COVID-19 vaccines between PLWH and people living without HIV (PLWoH), and the available literature on the risk of breakthrough infection by HIV status reports contradictory results [12, 17–19]. The aim of this study is to compare the risk of SARS-CoV-2 infection and severe COVID-19 in vaccinated individuals according to their HIV status in four Italian regions.

## Methods

### Study design and data sources

We conducted a matched cohort analysis to compare the risk of breakthrough SARS-CoV-2 infection and breakthrough infection leading to COVID-19 hospitalisation in PLWH and PLWoH. Data were obtained using TheShinISS, an R-based open-source statistical tool, developed by the National Institute of Health [20], that locally processes data collected and periodically updated from regional health care databases according to an ad hoc, study-tailored, Common Data Model (CDM). Over the last years, TheShinISS framework has been employed in several large-scale observational studies exploring the association between several exposures and COVID-19 onset/prognosis as well as other drug and vaccine-related research topics. It is currently maintained by a collaborative research network [21–26].

Subject characteristics were retrieved from several routinely collected regional healthcare claims databases. Demographic, clinical and vaccination variables were obtained from: (a) the COVID-19 vaccination registry which holds individual information on each vaccine administration (e.g., product, date of administration and doses for all vaccinated subjects); (b) the COVID-19 surveillance system which collects individual information on all notified SARS-CoV-2 infections and related outcomes; and (c) the healthcare population registry used to identify information on age, sex and vital status (causes of death are not recorded in this registry). HIV status and information on comorbidities of the study subjects in the period preceding vaccination were obtained from the following sources of data: (a) hospital discharge databases; (b) pharmacy claims; and (c) copayment exemptions databases. Regional claims data were locally transformed into a study specific CDM and locally processed using TheShinISS. All databases were linked deterministically using a unique regional individual identifier. Finally, regional pseudonymized datasets were provided to the National Institute of Health for centralized analyses, in compliance with EU General Data Protection Regulation. More detailed information about TheShinISS can be found online [20].

### Study population and period of study

We used data from four Italian regions that uploaded the required data to TheShinISS: Lombardy, Veneto and Emilia-Romagna (northern Italy); and Lazio from central Italy. These regions represent 42% of the total adult resident population in Italy. We investigated the risk of SARS-CoV-2 infection and COVID-19 hospitalisation by HIV status in adults aged ≥ 18 years who had completed the primary vaccination cycle with two doses of mRNA vaccines at least 7 days earlier (hereafter referred as fully vaccinated). We excluded individuals with a previously notified SARS-CoV-2 infection, those who were not completely vaccinated by the end of the study period and those diagnosed with rheumatoid arthritis - as they are also at risk of immune dysfunction.

The start of the study period was January 24, 2021, (28 days after the start of vaccination campaign on 27 December 2020) and the end of the study was September 21, 2021, seven days after the third booster dose was approved in Italy for priority groups (including PLWH) [27]. It Italy, the vaccination campaign prioritised some population groups according to their risk of SARS-CoV-2 infection/severe COVID-19. PLWH were in the second highest risk group, after HCWs, Long Term Care residents and persons aged 80 + years, and alongside those aged 60–79, those with severe comorbidities and school staff [28]. During the study period, the alpha (B.1.1.7) variant was dominant in Italy until July, when the delta (B.1.617.2) variant became dominant and remained so until the end of the study period [29].

### Outcomes and exposure

We measured two outcomes: time to SARS-CoV-2 infection, defined as a positive case of SARS-CoV-2 diagnosed through PCR or antigen test, and time to a SARS-CoV-2 infection that resulted in COVID-19 hospitalisation.

We classified as hospitalised cases any infection resulting in COVID-19 related hospitalisation within four weeks since diagnosis. The COVID-19 Italian surveillance system records only the hospitalisations presenting with clinical manifestations of the respiratory tract or other organs directly associated to SARS-CoV-2 infection.

The exposure variable was HIV status. We classified as PLWH those who had received: (a) a diagnosis of HIV identified through hospital admission, coded using International Classification of Disease, 9th Revision, Clinical Modification (ICD-9-CM code of HIV: 042); and/or (b) a prescription of antiviral drugs identified through pharmacy claims using Anatomical Therapeutic Chemical (ATC) classification system (codes of antiviral drugs: J05AE, J05AF, J05AG, J05AR); and/or (c) a notified copayment exemption for HIV infection identified through copayment exemptions database (code of HIV infection: 020).

### Statistical analysis

For each day of the study period, we matched (without replacement) a PLWH with four PLWoH. We carried out exact matching by ten-year age groups (from 18 to 29 years to > 80 years of age), sex, region of residence, calendar week of second dose administration and vaccine brand (Comirnaty/BNT16b2 or Moderna/mRNA-1273); and a propensity score matching for the Charlson Index, the number of drug prescriptions in the last year and specific comorbidities, choosing the four nearest neighbours. Details of the matching variables, alongside pre and post matching results can be found in the Supplementary Material 1.

We described the baseline characteristics of the matched pairs and of the population where they were drawn from, using counts with percentages and medians with interquartile range (IQR).

In outcomes analysis, follow-up started on the day each person was fully vaccinated (8th day post second dose) and ended on the day of testing positive for SARS-CoV-2 infection, the day of death (any cause) or at the end of the study period, whichever came first. We then computed cumulative probability curves of SARS-CoV-2 infection and SARS-CoV-2 infection leading to COVID-19 hospitalisation over time since full vaccination using the Kaplan-Meier estimator and the log-rank test to evaluate differences by HIV status. We compared risks in fully vaccinated according to HIV status using risk differences and ratios, taking PLWoH as the reference group. We also analysed outcomes stratifying by sex, age group (18–59 and 60+) and time since full vaccination (0-119 days and 120–233 days). We calculated 95% confidence intervals (CI) using percentiles from non-parametric bootstrapping with 500 sampling repetitions. All analyses were carried out using R software (version 4.2.2) [30].

## Results

### Study population

**Fig. 1:**
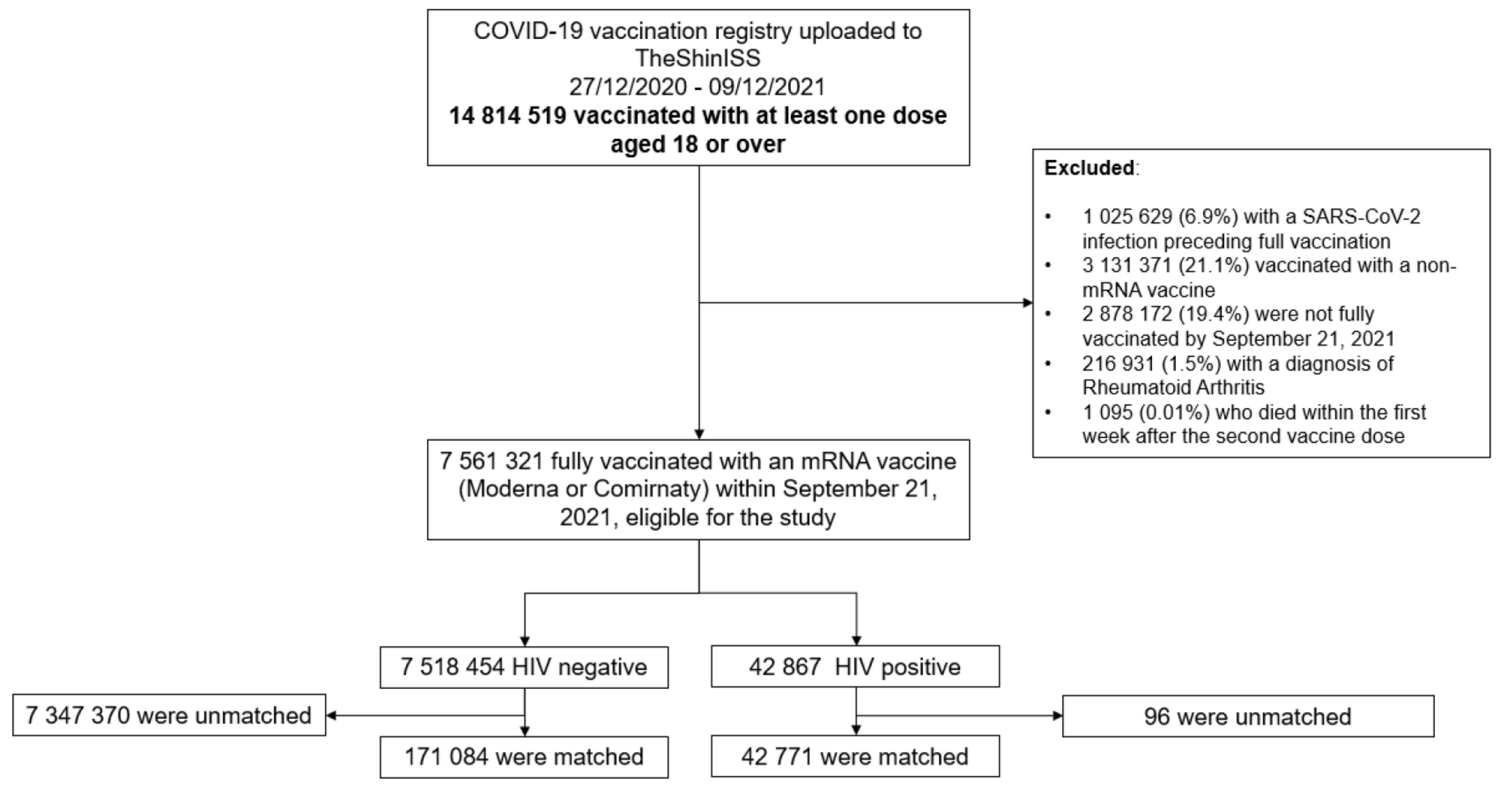
Selection of the population included in the analysis and matching

During the study period, 14 814 519 persons received at least one vaccine dose against COVID-19 in the included regions. Of these individuals, 7 561 321 were fully vaccinated with a mRNA vaccine by September 21, 2021 (Fig. 1). Among the 42 867 PLWH eligible for the study, 42 771 were matched with 171 084 PLWoH as controls. PLWoH were more frequently over 70 years of age, female and had a Charlson index of 0, compared with eligible PLWH (Table 1). The matched study groups were identical in the distribution by age, sex, vaccine brand and region of residence; and similar with respect to the Charlson index and all the other variables inserted in the propensity score (Supplementary Material 1).

**Table 1:** Characteristics of study participants

Variable	Eligible		Matched	
	PLWoH (*n* = 7 518 454)	PLWH (*n* = 42 867)	PLWoH (*n* = 171 084)	PLWH (*n* = 42 771)
Age distribution, n (%)				
18–29 yr	820,207 (10.9%)	883 (2.1%)	3532 (2.1%)	883 (2.1%)
30–39 yr	768,735 (10.2%)	3435 (8%)	13,684 (8%)	3421 (8%)
40–49 yr	1,103,103 (14.7%)	7590 (17.7%)	30,236 (17.7%)	7559 (17.7%)
50–59 yr	1,263,696 (16.8%)	15,255 (35.6%)	60,820 (35.5%)	15,205 (35.5%)
60–69 yr	1,090,120 (14.5%)	8702 (20.3%)	34,804 (20.3%)	8701 (20.3%)
70–79 yr	1,019,400 (13.6%)	5060 (11.8%)	20,240 (11.8%)	5060 (11.8%)
> 80 yr	1,453,193 (19.3%)	1942 (4.5%)	7768 (4.5%)	1942 (4.5%)
Females, n (%)	3,991,485 (53.1%)	11,891 (27.7%)	47,560 (27.8%)	11,890 (27.8%)
Charlson Index, n (%)				
0	6,595,391 (87.7%)	32,047 (74.8%)	122,426 (71.6%)	32,004 (74.8%)
1–2	824,723 (11%)	9519 (22.2%)	43,746 (25.6%)	9473 (22.1%)
3–4	89,738 (1.2%)	1184 (2.8%)	4526 (2.6%)	1177 (2.8%)
5+	8602 (0.1%)	117 (0.3%)	386 (0.2%)	117 (0.3%)
Vaccine brand, n (%)				
Cominarty	6,457,863 (85.9%)	34,507 (80.5%)	138,028 (80.7%)	34,507 (80.7%)
Moderna	1,060,591 (14.1%)	8360 (19.5%)	33,056 (19.3%)	8264 (19.3%)
Region of residence, n (%)				
Veneto	873,087 (11.6%)	4460 (10.4%)	17,836 (10.4%)	4459 (10.4%)
Lazio	1,058,326 (14.1%)	5495 (12.8%)	21,600 (12.6%)	5400 (12.6%)
Lombardia	4,639,116 (61.7%)	27,260 (63.6%)	109,040 (63.7%)	27,260 (63.7%)
Emilia-Romagna	947,925 (12.6%)	5652 (13.2%)	22,608 (13.2%)	5652 (13.2%)

### Relative and absolute risk of breakthrough infection and COVID-19 hospitalisation

**Fig. 2:**
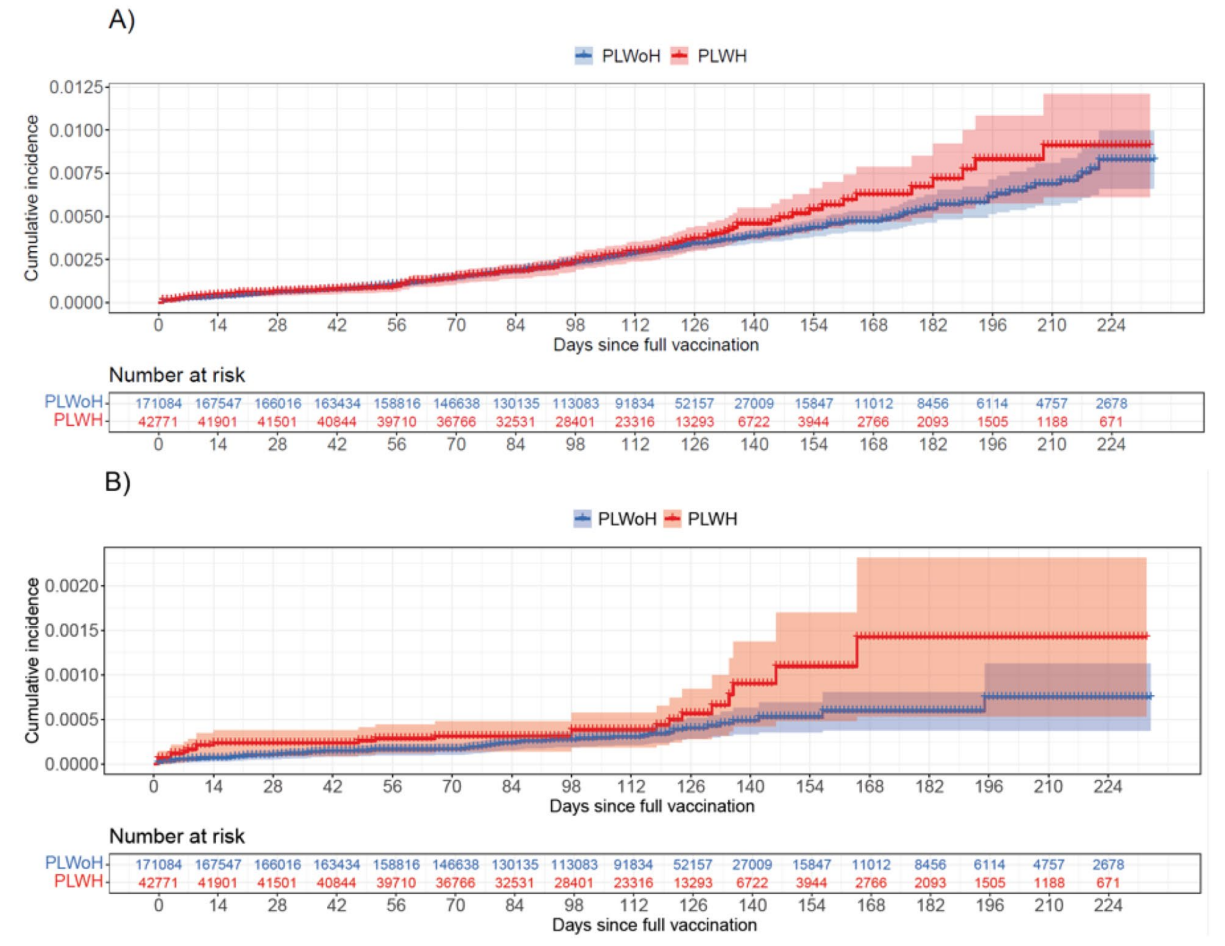
Cumulative incidence of SARS-CoV-2 breakthrough infections (**A**) and COVID-19 breakthrough hospitalisations (**B**) in patients living with HIV (PLWH) and in patients living without HIV (PLWoH). Shaded areas indicate 95% confidence intervals and plus signs censored data

The median duration of follow-up after full vaccination was 114 days (interquartile range, 85–130). Incidence of SARS-CoV-2 infection after full vaccination was similar in PLWoH and in PLWH for the entire follow-up period (Fig. 2, A), with a log rank test p equal to 0.3. Overall, 137 SARS-CoV-2 infections were detected in PLWH (91.10 cases per 10 000 person-days) and 497 in PLWoH (82.83 cases per 10 000 person-days) corresponding to an estimated rate ratio (RR) of 1.10 (95%CI: 0.80 to 1.53) and a risk difference between PLWH and PLWoH of 8.28 infections per 10 000 person-days (95% CI: -18.43 to 40.29) (Table 2). We did not observe significant differences in risk by sex or age. With regards to age, we found a 27% (95%CI: -12–79%) higher risk of infection in PLWH aged 18–59 compared to PLWoH of the same age group, which corresponds to an increase of 23.18 infections per 10 000 person-days (95%CI: -11.28 to 63.57). Conversely, we found a 33% (95%CI: -60% to + 11%) lower risk of breakthrough infection in PLWH aged 60+, corresponding to -24.51 infections per 10 000 person-days (95%CI: -58.56 to + 5.68). No differences were observed according to time since full vaccination. We carried out the same analysis using different matching ratios (i.e., 1:1, 1:2 and 1:3), obtaining similar results (Table S2 in Supplementary Material 2).

The cumulative probability of COVID-19 hospitalisation was higher in PLWH than in PLWoH, though confidence intervals of the Kaplan Meier estimator overlapped through the entire period (log rank p equal to 0.06) (Fig. 2, B). There were, overall, 23 COVID-19 hospitalisations in PLWH (14.24 events per 10 000 person-days) and 58 in PLWoH (7.51 events per 10 000 person-days) with a rate ratio of 1.90 (95%CI: 0.93 to 3.32) and a risk difference of 6.73 hospitalisations per 10 000 person-days (95%CI: -0.57 to 14.87) (Table 3). Males with HIV were 93% (95%CI: -21–307%) more likely to be hospitalised for COVID-19 following full vaccination, compared with males without HIV, which corresponds to an increase of 8.89 hospitalisations per 10 000 person-days (95%CI: -2.51 to 21.18). We also observed a non-significant increased risk in females with HIV, but of lower magnitude. PLWH aged 18–59 had a non-significant increased risk of hospitalisation compared with PLWoH of the same age, with a RR of 2.65 (95%CI: 0.53 to 6.83) and a risk difference of 5.29 hospitalisations per 10 000 person-days (95%CI: -1.75 to 13.55). Equally, no significant differences were observed in the 60 + age group. Finally, we did not observe significant difference in risk according to time since full vaccination, though the point estimate was higher in the second half of the study (120 to 233 days after full vaccination), when the relative increase in risk was 2.40 (95%CI: 0.81 to 8.91) and the absolute increase in hospitalisations 5.78 per 10 000 person-days (95%CI: -1.10 to 14.69).

**Table 2:** Estimated relative and absolute risk of SARS-CoV-2 infection in PLWH vs. PLWoH following mRNA full vaccination

Subgroup	PLWoH		PLWH		Rate Ratio [95% CI]	Risk Difference (per 10 000 individuals) [95% CI]
	*N*. events	Rate (per 10 000 person-days	*N*. events	Rate (per 10 000 person-days		
All	58	7.51	23	14.24	1.90 [0.93–3.32]	6.73 [-0.57-14.87]
Sex						
Males	46	9.53	18	18.42	1.93 [0.79–4.07]	8.89 [-2.51-21.18]
Females	12	3.64	5	5.89	1.62 [0.33–3.98]	2.25 [-2.78-7.8]
Age groups						
18–59	18	3.20	6	8.50	2.65 [0.53–6.83]	5.29 [-1.75-13.55]
60+	40	17.28	17	23.40	1.35 [0.42–3.75]	6.11 [-13.57-28.72]
Days since full vaccination						
0-119	48	8.59	16	13.48	1.57 [0.59–3.44]	4.89 [-3.81-17.67]
120–233	10	4.13	7	9.90	2.40 [0.81–8.91]	5.78 [-1.10-14.69]

**Table 3:** Estimated relative and absolute risk of COVID-19 hospitalisation in PLWH vs. PLWoH following mRNA full vaccination

Subgroup	PLWoH		PLWH		Rate Ratio [95% CI]	Risk Difference (per 10 000 individuals) [95% CI]
	*N*. events	Rate (per 10 000 person-days	*N*. events	Rate (per 10 000 person-days		
All	497	82.83	137	91.10	1.10 [0.80–1.53]	8.28 [-18.43-40.29]
Sex						
Males	344	80.60	103	101.02	1.25 [0.82–1.89]	20.42 [-14.29-64.67]
Females	153	88.16	34	71.33	0.81 [0.38–1.34]	-16.82 [-60.33-27.51]
Age group						
18–59	325	87.05	92	110.23	1.27 [0.88–1.79]	23.18 [-11.28-63.57]
60+	172	73.66	45	49.15	0.67 [0.40–1.11]	-24.51 [-58.56-5.68]
Days since full vaccination						
0-119	427	86.23	109	86.14	1.00 [0.76–1.32]	-0.09 [-21.46-25.43]
120–233	70	51.72	28	59.17	1.14 [0.68–1.85]	7.45 [-18.67-38.52]

## Discussion

### Summary of findings

We conducted a matched observational study in four Italian regions (accounting for 42% of the Italian population) to estimate the risk of SARS-CoV-2 infection and COVID-19 hospitalisation following full vaccination (two doses) with an mRNA vaccine in persons living with HIV compared to those living without HIV. We did not observe significant differences in the risk of infection following full vaccination between PLWH and PLWoH. We observed a higher risk of hospitalisation in PLWH compared with PLWoH, particularly in young males, but the increase in risk was not significant at the 95% level.

### Comparison with other studies in the literature and possible explanations

Previous observational studies comparing the risk of breakthrough infection in PLWH and PLWoH have found contradictory results. Two studies conducted in the US during a comparable time period (pre and post delta prevalence) found higher relative risks (28% and 33%) of breakthrough infection in PLWH compared with PLWoH [17, 19]. However, other observational studies, also in the US, found similar risk of breakthrough infections according to HIV status, though the precision of their estimates was low [18, 31]. Studies that have compared immunogenicity induced by COVID-19 vaccines in PLWH and in PLWoH have suggested that the humoral and cellular immune response elicited by vaccination depends upon the CD4 T-cell count. PLWH with CD4 T-cell counts above 500 cells/mm^3^ have a similar response with PLWoH [32], whereas those with less than 200 cells/ mm^3^ have a significantly lower immune response [16]. In Italy it is estimated that less than 10% of PLWH have CD4 counts below 200 cells/mm^3^, which could explain the similar risk of breakthrough infection found in both groups [33]. Though the risk of breakthrough infection was not significant, we observed higher point estimates in PLWH aged 18–59. We do not know if the lack of significance is due to a similar risk between PLWH and PLWoH in this age group or to a lack of power in our study. A previous study found that the risk of breakthrough infection, among adults living with HIV, decreased as age increases [19]; and suggested that it could be due to a higher adoption of protective behaviours, such as masks or social distancing, in the older population [34, 35]. Several studies have assessed the risk of severe COVID-19, including COVID-19 hospitalisations, in PLWH and PLWoH, with some finding an increased risk in PLWH [36, 37]. However, very few studies have compared the risk of hospitalisation between PLWH and PLWoH after vaccination. One observational study in the US found a similar risk in both groups, only finding an increased risk of hospitalisation in vaccinated PLWH with CD4 counts bellow 350 cells/µL [38]. These results coincide with our study, as we did not observe a significant increase in the risk of hospitalisation among PLWH. However, the high point estimates observed in young males could mean that these groups are at increased risk but that our study lacked power to detect significant differences. Equally, we observed a non-significant higher risk in PLWH in the latest period (four to seven months after full vaccination) which may suggest a quicker waning of vaccine-induced immunity in PLWH with respect to PLWoH, as other authors have suggested [39].

### Strengths and limitations

We linked well-established routine data sources to characterise the vaccinated population according to HIV status. Though these data sources are considered complete and accurate, it is possible that we did not capture all PLWH living in Italy, especially those who may not be in contact with healthcare services. We also classified participants according to HIV status at the beginning of the study period and we did not have information on new diagnosis during the period under study, though given the low incidence of HIV in 2021 [15], it is unlikely this had any effect on the estimates. Another limitation is that we were unable to categorise PLWH according to CD4 count and, thus, we could not assess the interaction between this factor and the risk of breakthrough infection. We carried out a rigorous matching for several characteristics which ensured that both groups (PLWH and PLWoH) were comparable. However, our estimates lacked precision due to the low number of events observed, particularly for COVID-19 hospitalisations, which reflects both the low prevalence of HIV in Italy and the high degree of protection induced by COVID-19 vaccination. Our analysis occurred in a period where underascertainment in Italy was lower than in omicron-prevalent periods [40], as testing availability was high and virus circulation was contained. However, if underascertainment occurred and was differential according to HIV status (e.g., PLWH being more aware of health risks may have been more likely to get tested) it could have biased our estimates probably towards an overestimation of the infection risk differences. Differential underascertainment could not bias our estimates of COVID-19 hospitalisation, as during the study period there was universal screening in Italy for all hospital admissions. It is possible, however, that although the surveillance expects only notifications of admissions due to COVID-19, some patients might have been notified as they were positive for SARS-Cov-2 even if they were admitted for reasons not related to COVID-19.If the likelihood of this misclassification was differential according to HIV status (e.g., PLWH being more likely to misclassified due to higher rates of hospital admission) it could have biased our risk estimates.

## Conclusions

We observed low rates of breakthrough SARS-CoV-2 infection and of COVID-19 hospitalisation both in PLWH and in PLWoH following full vaccination with mRNA vaccines in Italy during 2021, a year dominated by the alpha and delta variants. We did not observe a higher risk of SARS-CoV-2 breakthrough infection or of COVID-19 hospitalisation in PLWH.

### Electronic supplementary material

Below is the link to the electronic supplementary material.

Supplementary Material 1

## Data Availability

The datasets generated and/or analysed during the current study are not publicly available due privacy reasons. Some of the data use, such as data on vaccination or COVID-19 surveillance data are available at the aggregated level through the Ministry of Health and Istituto Superiore di Sanità websites.
