# Supplementary material for: Risk of breakthrough infection and hospitalisation after COVID-19 primary vaccination by HIV status in four Italian regions during 2021
Source: BMC Public Health. 2024 Jun 11;24:1569. doi: 10.1186/s12889-024-19071-y (PMC11165887; doi:10.1186/s12889-024-19071-y)
Supplement: Supplementary file 1 — Supplementary Material 1 [file 12889_2024_19071_MOESM1_ESM.docx]

**Supplementary Material**

Supplement to: Mateo-Urdiales A, Fabiani M et al. Risk of breakthrough infection and hospitalisation after COVID-19 primary vaccination by HIV status in four Italian regions during 2021.

**Supplementary Material 1.**

| **Table S1.** Demographic and health-related characteristics in people living with HIV and in people living without HIV in the eligible and matched populations included in the propensity score matching | | |
| --- | --- | --- |
| Variable | **PLWoH**  **(n = 171 084)** | **PLWH**  **(n = 42 771)** |
| Number of prescriptions in the last 12 months, mean (SD) | 21.49 (27.97) | 20.67 (24.93) |
| Number of prescriptions in the last 3 months, mean (SD) | 5.66 (7.66) | 5.37 (6.97) |
| NSAID prescribed in the last 3 months (%) | 10664 (6.2) | 2494 (5.8) |
| Estrogen/progesterone prescribed in the last 3 months (%) | 1895 (1.1) | 428 (1) |
| COPD (%) | 15759 (9.2) | 3600 (8.4) |
| Chronic pulmonary insufficiency (%) | 9674 (5.7) | 2244 (5.3) |
| Chronic Kidney Failure (%) | 5740 (3.4) | 1369 (3.2) |
| History of cancer (%) | 40413 (23.6) | 8765 (20.5) |
| Diabetes (%) | 24227 (14.2) | 4735 (11.1) |
| Lipid metabolism disorders (%) | 46589 (27.2) | 10073 (23.5) |
| Hematological disorders (%) | 38098 (22.3) | 8658 (20.2) |
| High Blood Pressure (%) | 70387 (41.1) | 16019 (37.5) |
| Liver Disorders (%) | 33957 (19.8) | 9225 (21.6) |
| Dementia (%) | 725 (0.4) | 181 (0.4) |
| Cystic fibrosis (%) | 11 (0.0) | 3 (0.0) |
| Peptic Ulcers (%) | 53063 (31) | 12031 (28.1) |
| Ulcerative colitis (%) | 1998 (1.2) | 331 (0.8) |
| Chronic infections (%) | 77759 (45.5) | 18934 (44.3) |

**Supplementary Material 2.**

| **Table S2**. Comparison of relative and absolute estimates of the risk of SARS-CoV-2 infection in PLWH vs PLWoH following mRNA full vaccination, according to matching ratio. | | | | | | |
| --- | --- | --- | --- | --- | --- | --- |
| **Subgroup** | **Matching 1:1** | | **Matching 1:2** | | **Matching 1:3** | |
|  | **Rate Ratio**  **[95% CI]** | **Risk Difference (per 10 000 individuals)**  **[95% CI]** | **Rate Ratio**  **[95% CI]** | **Risk Difference (per 10 000 individuals)**  **[95% CI]** | **Rate Ratio**  **[95% CI]** | **Risk Difference (per 10 000 individuals)**  **[95% CI]** |
| All | 1.26 [0.82-1.98] | 18.96 [-16.86-53.2] | 1.23 [0.89-1.74] | 16.78 [-11.09-47.74] | 1.11 [0.76-1.55] | 9.12 [-21.35-37.02] |
| Sex | | | | | | |
| Males | 1.66 [0.96-2.98] | 40.18 [-2.94-84.94] | 1.46 [0.92-2.39] | 31.87 [-7.61-74.91] | 1.29 [0.84-2] | 22.51 [-15.37-68.62] |
| Females | 0.76 [0.37-1.67] | -22.77 [-87.28-40.65] | 0.83 [0.42-1.48] | -14.23 [-59.16-32.75] | 0.79 [0.41-1.42] | -18.94 [-61.29-30.02] |
| Age group | | | | | | |
| 18-59 | 1.55 [0.98-2.67] | 38.94 [-1.97-82.72] | 1.48 [0.94-2.23] | 35.6 [-5.13-78.41] | 1.28 [0.87-1.85] | 24.23 [-12.61-63.5] |
| 60+ | 0.71 [0.4-1.42] | -20.27 [-62.7-18.98] | 0.6 [0.32-1.22] | -33.06 [-88.75-10.59] | 0.68 [0.43-1.24] | -23.12 [-60.28-12.21] |
| Days since full vaccination | | | | | | |
| 0-119 | 1.24 [0.9-1.74] | 16.85 [-7.41-43.61] | 0.96 [0.66-1.37] | -3.14 [-35.91-28.29] | 0.98 [0.71-1.29] | -2.18 [-29.66-22.91] |
| 120-233 | 1.34 [0.7-3.15] | 15.12 [-17.62-49.54] | 1.32 [0.73-2.46] | 14.36 [-15.73-45.98] | 1.16 [0.65-1.95] | 8.25 [-19.53-36.64] |
